# Impact of the Type of Anticoagulation Therapy on Long-Term Clinical Outcomes in Patients with Coronary Bifurcation Lesion and Atrial Fibrillation—Insights from the Bulgarian Bifurcation Registry

**DOI:** 10.3390/medicina60081294

**Published:** 2024-08-10

**Authors:** Niya Mileva, Dobrin Vassilev, Panayot Panayotov, Pavel Nikolov, Georgi Dimitrov, Kiril Karamfiloff, Gianluca Rigatelli, Robert J. Gil

**Affiliations:** 1 Medica Cor Hospital, 7000 Ruse, Bulgaria; dobrinv@gmail.com (D.V.); ppanayotov.medica@yahoo.com (P.P.); 2 Faculty of Public Health and Health Care, Ruse University “Angel Kanchev”, 7017 Ruse, Bulgaria; 3 Department of Cardiology, Pulmonology and Endocrinology, Medical Faculty, Medical University of Pleven, 5800 Pleven, Bulgaria; 4 Cardiology Department, Alexandrovska University Hospital, 1431 Sofia, Bulgaria; pavel.nikolov89@gmail.com (P.N.); jorinjo@abv.bg (G.D.); organic@abv.bg (K.K.); 5 Ospedali Riuniti Padova Sud, 35043 Padova, Italy; jackyheart71@yahoo.it; 6 National Medical Institute of Internal Affairs and Administration Ministry, 02-507 Warsaw, Poland; scorpirg@gmail.com

**Keywords:** coronary bifurcation, atrial fibrillation, percutaneous coronary revascularization, clinical outcome

## Abstract

*Background and Objectives*: Patients with atrial fibrillation and coronary artery disease represent a group with a greater risk of mortality. To evaluate patients with atrial fibrillation (AF) and a significant coronary bifurcation lesion and compare the clinical outcomes between the patients on anticoagulant treatment with Vitamin K antagonist (VKA) and those on direct anticoagulant (DOAC). *Materials and Methods*: This is a prospective study of patients with AF and stable coronary artery disease, who had evidence of a significant coronary bifurcation lesion. A log-rank test was used to assess the difference in mortality between patients taking VKA and those on DOAC. The primary endpoint was the incidence of all-cause and cardiovascular death at mid-term. *Results*: A total of 226 patients with AF and a significant bifurcation lesion were included. The mean age was 70.9 ± 9.2, and 70% were males. Of the patients, 123 (54.7%) were on VKA treatment, and 103 (45.3%) were taking DOAC. For a median follow-up time of 55 (39–96) months, overall mortality was 40%, whereas CV mortality was 31%. Both all-cause (28.2% versus 50.4%, *p* = 0.020) and CV death (12.7% versus 24.9%, *p* = 0.032) were significantly lower in patients taking DOAC versus those on VKA. In patients treated with PCI, CV mortality was significantly lower in patients taking DOAC (21.4% versus 40.5%, *p* = 0.032). VKA therapy was an independent predictor of cardiovascular death (HR 1.88; 95% CI 1.11–3.18; *p* = 0.01), together with chronic kidney disease (HR 1.81; 95% CI 1.13–2.92; *p* = 0.01). *Conclusions*: Treatment with DOAC in patients with atrial fibrillation and coronary bifurcation lesion was associated with significantly lower mortality independently of the treatment approach. VKA was an independent predictor of CV mortality.

## 1. Introduction

Globally atrial fibrillation (AF) is the most prevalent sustained cardiac arrhythmia in adults [1]. What is more, the incidence of AF is increasing, in accordance with the aging of the patient population and the acceleration of co-morbidity [2,3]. AF increases the risk of thromboembolic events and major cardiovascular adverse events, and it is one of the leading causes of morbidity and mortality [4]. Atrial fibrillation shares common risk factors with coronary artery disease (CAD); therefore, patients with CAD often have coexistent arrhythmia, and these subsets of patients frequently undergo percutaneous coronary intervention (PCI). Previous studies revealed that known AF is associated with a higher risk of in-hospital complications and mortality after PCI [5,6]. Patients with AF who require PCI pose a challenging clinical dilemma due to the need for combined antithrombotic therapy and the perpetual counteraction between thrombotic and bleeding risk [7]. Although there are plenty of published data on the advantage of direct oral anticoagulants (DOAC) over vitamin K antagonists (VKA) for stroke prevention in patients with AF [8,9,10,11] and despite the current clinical practice recommendations [4], triple therapy with VKA is still broadly used in clinical practice [12]. These findings highlight the importance of AF as an independent risk factor for poor clinical outcomes after PCI.

On the other hand, coronary bifurcation lesions represent a clinical and interventional challenge. Even though in the last years considerable progress in the treatment of coronary bifurcation lesions has been achieved [13,14], these patients are still representing a subset of CAD associated with higher complication rates and generally worse clinical outcomes [15,16,17].

Despite the frequency of patients with atrial fibrillation and coronary artery disease, there are deficient data from contemporary practice describing the impact of the type of anticoagulation in patients with AF and coronary bifurcation lesions. The aims of the current study were to analyze the type of anticoagulation therapy in the high-risk group of patients with coronary bifurcation lesions and atrial fibrillation and its effect on mortality at long-term follow-up.

## 2. Materials and Methods

### 2.1. Study Design and Patient Selection

This is a multi-center single-country investigator-initiated prospective registry of patients with coronary bifurcation stenoses starting from January 2013—Bulgarian Bifurcation Registry. The inclusion criteria for the current analysis were patients with stable coronary artery disease who had angiographic evidence of a significant coronary bifurcation lesion and any form of atrial fibrillation. Patients with acute coronary syndrome, left main stenosis, non-cardiac co-morbid conditions with a life expectancy of less than one year were excluded. All patients were managed in accordance with the Declaration of Helsinki. The study protocol was performed in accordance with the Ethics Committee of our institution. All patients signed written informed consent to be included in the registry.

### 2.2. Definition of Endpoints

Significant bifurcation lesion was considered any angiographic bifurcation lesion in a native coronary artery with a diameter ≥ 2.5 mm and ≤4.5 mm and SB diameter ≥ 2.0 mm and percentage diameter stenosis ≥50% in the main vessel. Angiographic success was defined as the end procedural MV (main vessel) percent diameter stenosis (%DS) < 20% and SB (side branch) diameter stenosis <70% without significant dissection and flow impairment [18]. The periprocedural myocardial infarction was defined according to 4th Universal Definition of Myocardial Infarction [16]. Major cardiovascular adverse events (MACE) were a composite of cardiovascular death, stroke, and non-fatal myocardial infarction. Cardiovascular death was defined as death with clearly determined cardiac origin or death from an unknown reason. Cerebrovascular disease was defined as a pathology in cerebral circulation such as a transitory ischemic attack, ischemic stroke, or hemorrhagic stroke. Chronic kidney disease was defined as eGFR < 60 mL/min. All patients were followed up by telephone contact and/or clinical visit at 30 days and then monthly for vital status through the insurance number in the National Insurance Institute.

### 2.3. Study Population

**Figure 1:**
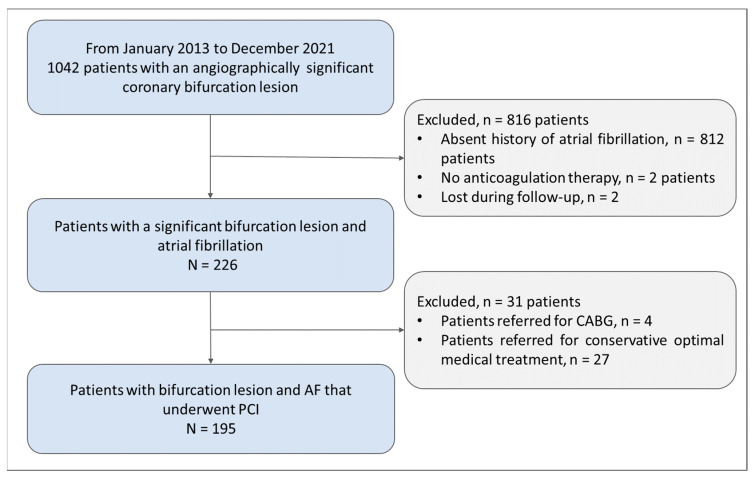
Flowchart. Patients included in the study.

From January 2013 to December 2021, 1042, patients with angiographic bifurcation lesions were included in the Bulgarian Bifurcation prospective registry. As shown in the study flowchart (Figure 1), 816 patients were excluded, with 812 patients lacking a history of AF, and 2 patients having AF, but with no indication for anticoagulation therapy. Moreover, 2 patients were lost during follow-up. The final study population consisted of 226 patients eligible for the main analysis.

### 2.4. Angiographic Analysis

All analyses were performed with dedicated QCA software (GE Healthcare, Boston, MA, USA) and additionally with MicroDicom QCA software, version 6.2, following the principles for coronary bifurcation stenosis analysis according to the Academic Research Consortium [14,15]. The minimal luminal diameter (MLD), reference vessel diameter (RVD), and %DS ([(RVD − MLD)/RVD] × 100) were measured for every segment of the bifurcation (i.e., proximal, and distal MV—pMV, dMV, and SB) pre-and post-intervention. Lesion length was measured from the proximal main vessel to the distal main branch (i.e., we considered beginning and ending points where hypothetically the stent will be implanted). All analyses were performed by two blinded and independent investigators (P.N. and G.D.), and in case of disagreement, a consensus was formed with additional analysis from the first author (D.V.).

### 2.5. Statistical Analysis

Patients were divided into two groups—those receiving therapy with direct oral anticoagulant (DOAC) and patients receiving vitamin K antagonist (VKA). Differences between groups were examined with paired or unpaired *t*-tests as appropriate, with normal distributions. Otherwise, the Wilcoxon sign-ranked test and Mann–Whitney U-tests were used. Chi-square tests were applied for qualitative data. Correlation analysis was performed with Pearson or Spearman test depending on the type of data. A Kaplan–Meier analysis with a log-rank test for between-group differences was also performed. Additionally, a multivariate Cox regression analysis, with backward elimination, was performed for the identification of independent predictors of all-cause death and cardiovascular death. The statistical differences are deemed significant if *p* < 0.05. All analyses were performed using R Studio version 4.0.3. (R Foundation for Statistical Computing, Vienna, Austria) Statistical Package for Social Sciences, version 23.0 (SPSS, PC version, Chicago, IL, USA).

## 3. Results

### 3.1. Clinical and Demographic Characteristics

The median age of the population was 72 (12) years, 70% (*n* = 157) were males, 27.4% (*n* = 62) were smokers, and 43% (*n* = 97) were diabetics. Additionally, 22.6% (*n* = 51) had a previous MI, and mean left ventricular ejection fraction (EF) was 52 ± 10%. The mean CHA2ds-VASc score was 3.2 ± 0.7 for males and 3.9 ± 1.1 for females. The mean HAS-BLED bleeding risk score was—2.4 ± 1.1. A total of 123 patients (54.7%) were on anticoagulation treatment with VKA, whereas 102 (45.3%) received a DOAC. Patient demographic and clinical characteristics and differences between groups on VKA and DOAC are presented in Table 1. In general, characteristics were well balanced between the two groups. However, patients receiving DOAC therapy had a higher frequency of oncologic disease—9.7% (*n* = 10) vs. 4.9% (*n* = 6), *p* = 0.002 compared to patients on VKA. Furthermore, patients on DOAC had a higher prevalence of cerebrovascular disease (26.2% vs. 18.7%, *p* = 0.011), but a smaller proportion of patients on DOAC had chronic kidney disease (36.9% vs. 48.8%, *p* = 0.004). Most patients were treated with a PCI–195 (85.8%), whereas 27 patients (11.9%) were left on conservative treatment with optimal medical therapy, and only 4 patients (1.8%) were referred to cardiac surgery.

### 3.2. PCI Group

For the 195 patients who underwent percutaneous coronary intervention, the target vessel was LAD/diagonal in 128 patients (66%), LCx—41 patients (21%), and RCA—in 26 (13%) patients. Eighty-four (41%) of the patients were on VKA treatment, and 111 (57%) were on DOAC. Clopidogrel was the main antiplatelet agent used in 173 (89%) of patients, whereas 17 (11%) patients received ticagrelor. Patients in the DOAC group had significantly higher grade of %DS (62.5 ± 28.2 vs. 80.0 ± 14.1, *p* = 0.001) and longer lesion length 31.6 ± 17.3 mm vs. 35.0 ± 16.7 mm, *p* = 0.016. Demographic and procedural characteristics of the PCI subgroup are described in Table 2.

### 3.3. Clinical Outcomes

**Figure 2:**
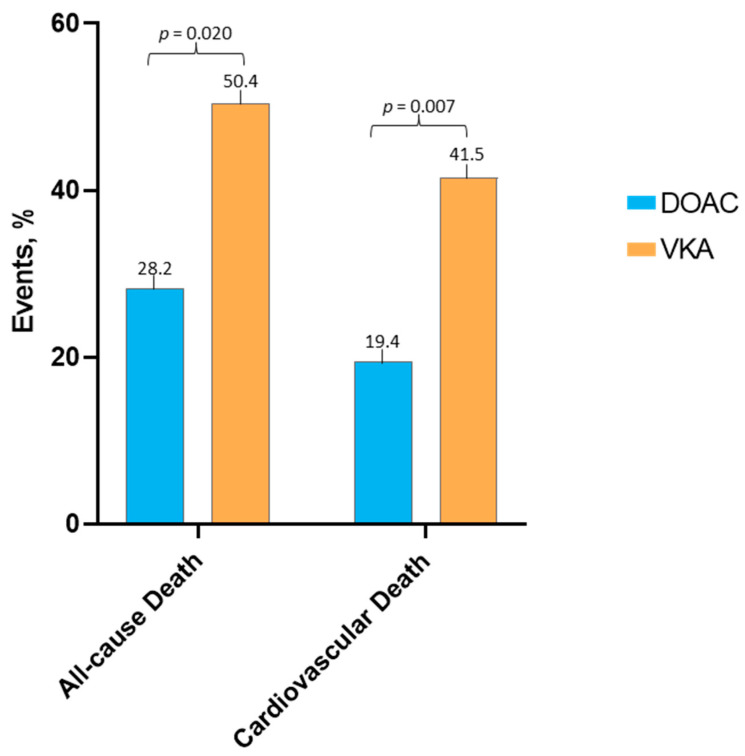
Clinical outcomes according to the type of anticoagulant therapy.

For a median follow-up time of 55 (39–96) months, overall mortality was 40% (*n* = 91), whereas CV death occurred in 31% (*n* = 71) of the patients. All-cause death was significantly lower in patients taking DOAC—28.2% (*n* = 29) versus 50.4% (*n* = 62) in patients on VKA, *p* = 0.020 (Figure 2).

**Figure 3:**
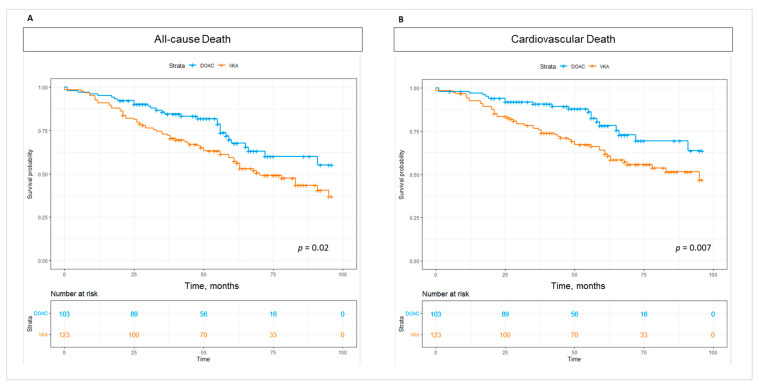
Whole group Kaplan–Meier survival curves—(**A**) all-cause mortality; (**B**)—cardiovascular mortality.

Furthermore, CV death was significantly lower in patients taking DOAC—19.4% (*n* = 20) versus 41.5% (*n* = 51) in patients on VKA, *p* = 0.007, (Figure 3).

**Figure 4:**
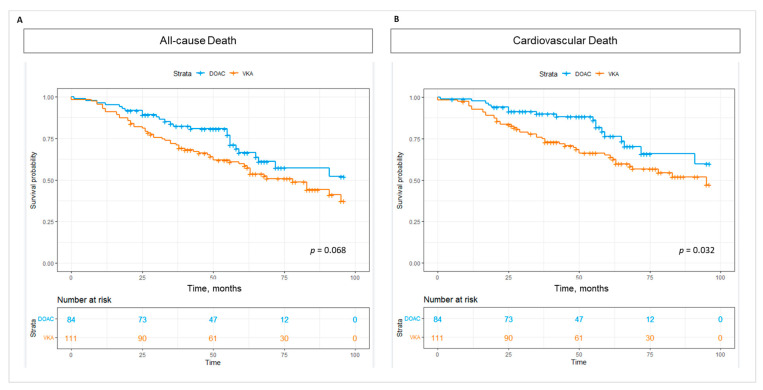
PCI group Kaplan–Meier survival curves—(**A**). all-cause mortality; (**B**)—cardiovascular mortality.

When analyzing the PCI group, all-cause mortality was numerically lower in patients on DOAC (26 (30.9%) versus 55 (49.5%) in patients on VKA, *p* = 0.068). Cardiovascular mortality was significantly lower in patients taking DOAC—18 (21.4%) versus 45 (40.5%) in patients on VKA, *p* = 0.032, Figure 4.

### 3.4. Predictors of Mortality

**Figure 5:**
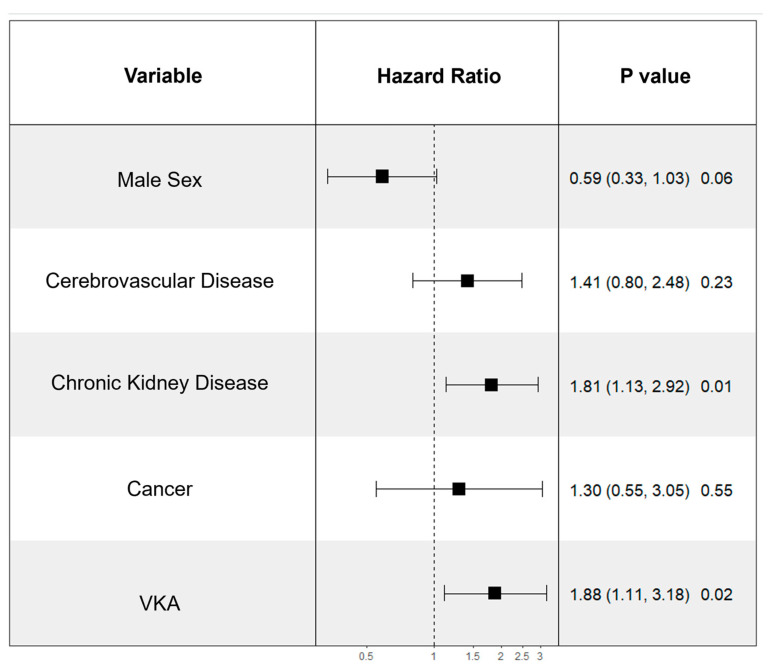
Forest plot illustrating Cox regression analysis with independent predictors of CV Death.

The following factors that were found significantly different between the two groups were included in multivariate Cox regression analysis to identify predictors of CV death: sex, cerebrovascular disease, chronic kidney disease, presence of cancer, and the type of anticoagulant therapy. In Cox regression analysis, VKA therapy remained an independent predictor of cardiovascular death (HR 1.88; 95% CI 1.11–3.18; *p* = 0.01), together with chronic kidney disease (HR 1.81; 95% CI 1.13–2.92; *p* = 0.01, Figure 5).

### 3.5. Bleeding Risk Stratification

The mean HAS-BLED bleeding risk score for the whole population was—2.4 ± 1.1. Of the patients, 27% (*n* = 61) had a bleeding risk score of ≥3 and were classified as the high bleeding risk group, whereas 73% (*n* = 165) had a HAS-Bled score < 3 and were classified into mild/moderate bleeding risk groups, Table 3.

Patients in the group with high bleeding risk were significantly older (75.9 ± 9.4 vs. 67.3 ± 9.4, *p* = 0.015), had a higher frequency of chronic kidney disease (54% vs. 39%, *p* < 0.001), had higher rates of previous myocardial infarction (24% vs. 19%, *p* < 0.026) and previous PCI (47 vs. 38%, *p* = 0.012). Moreover, patients in the high bleeding risk group received higher rates of CABG (3% vs. 0.8%, *p* = 0.019) and PCI (95 vs. 83%, *p* = 0.010) and lower rates of conservative treatment (8% vs. 13%, *p* = 0.006) when compared with the low bleeding risk group.

**Figure 6:**
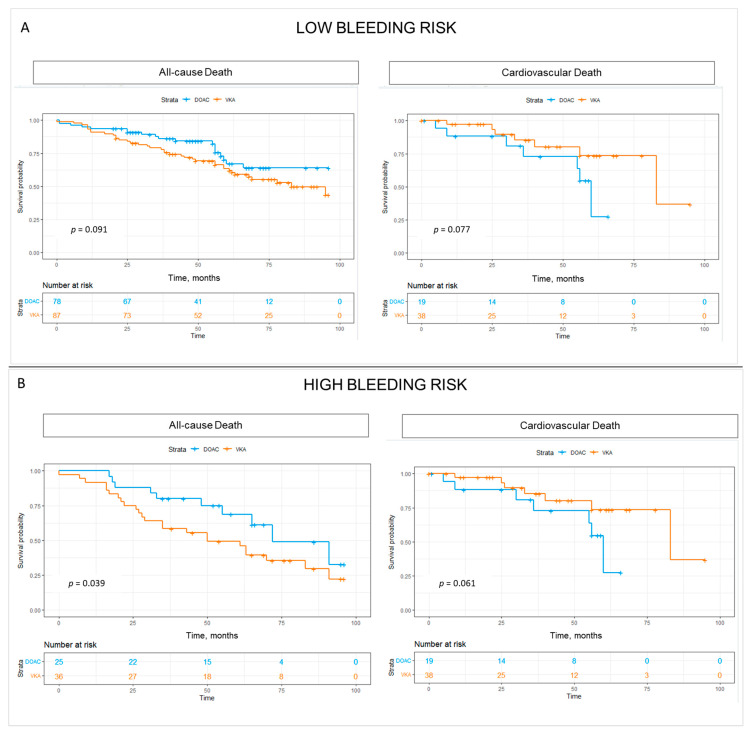
Kaplan–Meier survival curves comparing the rates of all-cause mortality and cardiovascular mortality in patients with (**A**) low/moderate bleeding risk and (**B**) high bleeding risk according to HAS-BLED score.

All-cause mortality for the whole low/moderate bleeding risk group was 34.5% (*n* = 57). Patients receiving VKA had significantly higher rates of all-cause death 41.5% (*n* = 51) versus 19.4% (*n* = 20) of patients on DOAC, *p* = 0.091. Cardiovascular mortality in the low/moderate bleeding risk group was 26% (*n* = 43) in total. Rates of cardiovascular death were numerically higher in the VKA group with 35.6% (*n* = 31) versus 15.4% (*n* = 12) in patients on VKA, *p* = 0.77, Figure 6A.

For the whole high bleeding risk group, all-cause mortality was 55.7% (*n* = 34). Patients receiving VKA had significantly higher rates of all-cause death 66.7% (*n* = 24) versus 40% (*n* = 10) of patients on DOAC, *p* = 0.039. Cardiovascular mortality in the high bleeding risk group was 46% (*n* = 28) in total. Rates of cardiovascular death were numerically higher in the VKA group with 55.6% (*n* = 20) versus 32% (*n* = 8) in patients on VKA, *p* = 0.061, Figure 6B.

## 4. Discussion

To the best of our knowledge, this is the first study to assess the impact of the type of anticoagulation therapy in patients with atrial fibrillation and a coronary bifurcation lesion. The main findings of our manuscript are the following: (i) Atrial fibrillation is highly prevalent among patients with coronary bifurcation lesions; (ii) DOAC therapy was associated with significantly lower all-cause and cardiovascular mortality when compared to a VKA in patients with coronary bifurcation lesions, independently of the type of treatment approach regarding the coronary artery disease; (iii) The effect of lower CV mortality in patients with DOAC was preserved in the subgroup with PCI approach; (iv) VKA therapy was an independent predictor of CV mortality.

These data are important for several reasons. As far as we know, this is the first study to describe the prevalence of AF in patients with coronary bifurcation lesions in contemporary clinical practice, which is itself an important observation. We found that a history of AF was present in almost 22% of the patients included in the Bulgarian Bifurcation registry, highlighting the omnipresence of this pathology among patients with coronary artery disease. Importantly, this number is significantly higher than the general population prevalence of AF reported in previously published data (5% to 11%) [19,20,21].

When considered independently, both atrial fibrillation and coronary artery disease bear risks for the patients and pose a significant burden to healthcare systems [22]. Furthermore, when combined, these entities represent an even higher overall population risk [6]. The latter fact is accentuated by the results from our study that reveal considerably high all-cause and CV mortality for the whole group of patients with atrial fibrillation and a coronary bifurcation lesion.

More importantly, patients in the high bleeding risk group exhibited remarkably high all-cause (56%) and CV mortality (46%) at a long-term follow-up. The effect of statistically lower death rates in the group on DOAC persevered for patients with a HAS-BLED score ≥ 3, whereas the difference in mortality was less significant in patients with a HAS-BLED score < 3. This finding accentuates the importance of the choice of drug agent in the population with the highest risk. Resources should be targeted to further evaluate the factors accountable for this association and to reduce morbidity and mortality in this high-risk subgroup.

Interestingly, the exaggerated risk of mortality among patients taking VKA was noted across the whole spectrum of treatment approaches and should be taken into account when considering risk estimation. We found that patients taking DOAC in the PCI subgroup had more severe coronary artery disease characterized by significantly longer lesions and greater %DS. Interestingly, despite this more advanced atherosclerosis, these patients had significantly lower all-cause and cardiovascular mortality.

It is noteworthy to comment on the rather high proportion of patients on VKA therapy. According to the current clinical practice guidelines, DOAC is the first anticoagulant of choice, whereas VKAs are left as a second-line drug or preferred only in specific clinical scenarios [4,23]. However, it is important to note that the current analysis evaluates patients included in the registry more than 10 years ago when DOACs were not as deeply implicated in contemporary current practice as to the current date [24,25].

Our results revealed that therapy with VKA was the most powerful independent predictor of CV mortality in a model including well-proven risk factors such as the presence of neoplasm and chronic kidney disease. Even though there is an abundance of evidence proving the benefit of DOAC versus VKA in patients with atrial fibrillation [8,9,10,11] and those undergoing PCI [7,26], it is crucial to identify the importance of anticoagulant choice in this very high-risk subgroup of patients with atrial fibrillation and coronary bifurcation lesions.

### Limitations

Limitations of this study include that this is an analysis of observational, nonrandomized data. This report includes data from one country in Eastern Europe, whose population is notorious for its high health risk. Therefore, it is unknown to what degree population variations may influence the results. The study was not powered to assess for specific cause of death, neither for bleeding nor thrombotic events that may have occurred. However, the study reveals a strong and consistent association between VKA and mortality. The study protocol was developed with an intention to treat analysis, assessing the patient outcome according to the treatment at the moment of discharge. Changes in treatment plans were not taken into account in the current analysis. Last but not least, the CHA2DS2-VASc score is an important tool for thrombotic risk evaluation that should be considered in the prognostic risk stratification of patients with atrial fibrillation. Although the mean value of the score was reported in our study, we have not specifically evaluated its prognostic value in the current population.

## 5. Conclusions

DOAC therapy in patients with significant coronary bifurcation lesions is associated with lower all-cause and cardiovascular mortality when compared to a VKA, independently of the type of treatment approach regarding coronary artery disease. The effect of lower CV mortality in patients with DOAC was preserved in the subgroup with the PCI approach. VKA therapy was an independent predictor of CV mortality.

## Figures and Tables

**Table 1:** Whole group—demographic characteristics.

Patient Characteristics	All N = 226	VKA N = 123	DOAC N = 103	*p*-Value
Age, years (median (IQR))	72 (12)	72 (10)	71 (13)	0.212
Males, *n* (%)	157 (69.5)	90 (73)	67 (65)	0.021
Hypertension, *n* (%)	223 (98.7)	121 (98.4)	102 (99)	0.179
Hyperlipidemia, *n* (%)	208 (92)	111 (90.2)	97 (94.2)	0.541
Diabetes, *n* (%)	97 (43)	54 (43.9)	43 (41.7)	0.753
Chronic kidney disease *n* (%)	98 (43.4)	60 (48.8)	38 (36.9)	0.004
Cancer, *n* (%)	16 (7.1)	6 (4.9)	10 (9.7)	0.004
Smoking, *n* (%)	62 (27.4)	35 (28.5)	27 (26.2)	0.485
Cerebrovascular disease, *n* (%)	50 (22.1)	23 (18.7)	27 (26.2)	0.011
Peripheral artery disease, *n* (%)	29 (12.8)	39 (12)	11 (10.7)	0.749
Previous myocardial infarction, *n* (%)	51 (22.6)	27 (22)	24 (23.3)	0.134
Previous PCI, *n* (%)	101 (44.7)	53 (43)	48 (46.6)	0.611
COPD, *n* (%)	37 (16.4)	21 (17.1)	16 (15.6)	0.262
LV EF < 50%, *n* (%)	80 (35)	42 (34)	36 (35)	0.601
Clopidogrel, *n* (%)	192 (85)	108 (87.8)	84 (81.6)	0.403
Aspirin, *n* (%)	206 (91.2)	116 (94.3)	90 (87.4)	0.212
Ticagrelor, *n* (%)	18 (8)	10 (8.1)	8 (7.8)	0.029
Treated with PCI, *n* (%)	195 (86.2)	111 (90.3)	84 (81.6)	0.052
Treated with CABG, *n* (%)	4 (1.8)	1 (0.8)	3 (2.9)	0.021
Conservative treatment, *n* (%)	27 (12)	11 (8.9)	16 (15.5)	0.013
SYNTAX score, mean ± sd	11.1 ± 5.6	10.8 ± 5.4	11.2 ± 5.8	0.397

**Table 2:** PCI group—demographic characteristics.

Patient Characteristics	All N = 195	VKA N = 111	DOAC N = 84	*p*-Value
Intervention vessel: o LAD, *n* (%) o LCx, *n* (%) o RCA, *n* (%)	o 128 (66) o 41 (21) o 26 (13)	o 8 (70) o 19 (17) o 14 (13)	o 55 (65) o 19 (23) o 10 (12)	o 0.212 o 0.212 o 0.212
Scopic time, min (mean ± sd)	21 ± 10	21 ± 9.4	22 ± 11	0.931
Contrast, ml (mean ± sd)	290 ± 104	294 ± 99.8	284 ± 109	0.179
SYNTAX score (mean ± sd)	11.4 ± 5.6	11.2 ± 5.4	11.7 ± 6.0	0.503
Clopidogrel, *n* (%)	173 (89)	100 (90)	73 (87)	0.311
Aspirin, *n* (%)	186 (95)	108 (97)	78 (93)	0.100
Ticagrelor, *n* (%)	17 (9)	9 (8)	8 (10)	0.089
%DS, mean ± sd	65.4 ± 26.8	62.5 ± 28.2	80.0 ± 14.1	0.001
Lesion length, mm (mean ± sd)	32.9 ± 17.1	31.6 ± 17.3	35.0 ± 16.7	0.016
Number of stents implanted, mean ± sd	1.6 ± 0.99	1.6 ± 0.99	1.6 ± 0.98	0.824
Stent diameter, mm (mean ± sd)	3.1 ± 0.3	3.2 ± 0.3	3.1 ± 0.4	0.613

**Table 3:** Difference in patients’ characteristics in groups with low/moderate and high bleeding risk.

Patient Characteristics	All N = 226	HAS-BLED < 3 N = 165	HAS-BLED ≥ 3 N = 61	*p*-Value
Age, years (mean ± sd)	70.9 ± 9.2	67.3 ± 9.0	75.9 ± 9.4	**0.015**
Males, *n* (%)	157 (69.5)	112 (68)	41 (70)	0.323
Hypertension, *n* (%)	223 (98.7)	160 (99)	60 (98)	0.519
Hyperlipidemia, *n* (%)	208 (92)	111 (90.2)	97 (94.2)	0.541
Diabetes, *n* (%)	97 (43)	54 (43.9)	43 (41.7)	0.753
Chronic kidney disease *n* (%)	98 (43.4)	64 (39)	33 (54.2)	**<0.001**
Cancer, *n* (%)	16 (7.1)	4 (6)	13 (8)	0.108
Smoking, *n* (%)	62 (27.4)	46 (28)	16 (26)	0.485
Cerebrovascular disease, *n* (%)	50 (22.1)	33 (20)	14 (23)	0.231
Peripheral artery disease, *n* (%)	29 (12.8)	20 (12)	18 (11)	0.695
Previous myocardial infarction, *n* (%)	51 (22.6)	31 (19)	15 (24)	**0.026**
Previous PCI, *n* (%)	101 (44.7)	62 (38)	29 (47)	**0.012**
COPD, *n* (%)	37 (16.4)	28 (17)	9 (16)	0.461
LV EF < 50%, *n* (%)	80 (35)	55 (33)	22 (36)	0.109
Treated with PCI, *n* (%)	195 (86.2)	137 (83)	58 (95)	**0.010**
Treated with CABG, *n* (%)	4 (1.8)	1 (0.8)	2 (3)	**0.019**
Conservative treatment, *n* (%)	27 (12)	22 (13)	5 (8)	**0.006**
SYNTAX score, mean ± sd	11.1 ± 5.6	10.9 ± 5.2	11.3 ± 5.9	0.471

Values in bold are statistically significant.

## Data Availability

The data presented in this study are available upon request from the corresponding author.
